# Editorial: Insights into the use of deep brain stimulation as a treatment for Parkinson's disease and related conditions

**DOI:** 10.3389/fnins.2023.1322091

**Published:** 2023-11-15

**Authors:** Zhi Liu, Yi Zhou, Ya Gao, Xiaofei Hu

**Affiliations:** ^1^Neurosurgery Department, The First Affiliated Hospital, Army Medical University, Chongqing, China; ^2^Department of Neurology, 980 Hospital of PLA Joint Logistics Support Forces, Shijiazhuang, Hebei, China; ^3^Neuroscience Institute, Dietrich College of Humanities and Social Sciences, Carnegie Mellon University, Pittsburgh, PA, United States; ^4^Department of Nuclear Medicine, Southwest Hospital, Army Medical University, Chongqing, China

**Keywords:** deep brain stimulation, Parkinson's disease, gait, dyskinesia, akinesia

## Introduction

Parkinson's Disease (PD), the second most prevalent progressive neurodegenerative disorder, manifests through motor symptoms, including resting tremors, rigidity, bradykinesia, and postural instability (Tolosa et al., 2021). Over the last 50 years, levodopa medication replacement therapy has reigned as the cornerstone of PD treatment (Romito et al., 2009). Nevertheless, as the disease advances, the medication's efficacy wanes, and extended use may precipitate complications such as dyskinesia and fluctuating responses. In the latter part of the 1990s, Benabid et al. uncovered that Deep Brain Stimulation (DBS) could alleviate PD's motor symptoms (Limousin et al., 1995). Owing to its minimally invasive, reversible, and adjustable nature, DBS has surfaced as an effective surgical intervention for PD. Recent years have ushered in notable advancements in both the fundamental research and clinical application of DBS. This editorial offers insights into employing DBS as a PD treatment.

### Progressive understanding of STN-DBS

The Subthalamic Nucleus (STN), recognized as the most prevalent DBS target in PD, enacts its therapeutic effects likely through widespread changes in neural activity across the brain. Animal research introduces nuances, allowing the activity of neuronal subpopulations to be selectively recorded and manipulated (Gittis and Yttri, 2018). With an optogenetic approach, researchers dissected out the excitatory afferents to STN alone, specifically fibers from the primary motor cortex, which is sufficient to induce therapeutic effects (Gradinaru et al., 2009). Nevertheless, electrophysiological observations during STN-DBS in monkeys revealed that the recruitment of cortex through antidromic signals diminished after 4 h of stimulation, without any accompanying reduction in therapeutic effectiveness (Johnson and Wang, 2020), implying that cortical activation is not a prerequisite for DBS's therapeutic efficacy. Conversely, focusing on local changes within the STN, the pathological firing patterns can also forecast the therapeutic efficacy of DBS (Dorval et al., 2010; Mcconnell et al., 2012, 2016). High-frequency local optogenetic stimulation (>75 Hz) that disrupts this firing pattern was adequate to enhance movement (Schor et al., 2022), while low-frequency or continuous stimulation, unable to alter the firing pattern, did not restore movement.

### Cell-type specific discoveries to persistent DBS efficacy

Historically, the significance of cell-type specificity in the pathological circuit has been marginally addressed in disease therapeutics. However, the cellular heterogeneity within the Globus Pallidus externa (GPe) has yielded intriguing findings. Interventions targeting distinct neuronal subgroups within the GPe manifest varied impacts on behavior (Cui et al., 2021; Isett et al., 2023). Optogenetic interventions that selectively elevated the activity of parvalbumin-expressing GPe neurons (PV-GPe) or suppressed the activity of lim homeobox 6-expressing GPe neurons (Lhx6-GPe) initiated a durable recovery of movement in bilaterally dopamine-depleted mice (Mastro and Zitelli, 2017). Spix et al. (2021) identified a frequency-dependent electrical stimulation paradigm to enact cell-type-specific neuromodulation in the GPe without resorting to optogenetics, with the therapeutic effect duration of this approach outpacing that achieved with traditional DBS protocols. While the frequency-dependent therapeutic effect has been clinically observed (Zapata Amaya et al.), further elucidation may arise from animal research.

### Efficacy of DBS in PD treatment

Both STN and internal globus pallidus (GPi) stimulations have proven notably effective in ameliorating core motor symptoms such as tremors, rigidity, and bradykinesia in advanced-stage PD patients (Tsuboi et al., 2020; Park et al., 2022). Porta et al. found that administering DBS treatment prior to initiating L-Dopa treatment exerts beneficial effects on motor symptoms and quality of life in early-stage PD. Liu et al.'s research found that individual substantia nigra (SN) susceptibility features, derived from radiomics, can predict global motor and rigidity outcomes post-STN-DBS in PD patients. An ongoing debate persists regarding the efficacy of STN-DBS in ameliorating axial symptoms in PD, such as gait, swallowing, speech, and balance issues (Collomb-Clerc and Welter, 2015; Guimarães and Cury, 2022). A recent meta-analysis (Schlenstedt et al., 2017) revealed that STN-DBS indeed improved gait characteristics (such as stride length and speed) and alleviated the freezing of gait in patients within 48 months after electrode implantation during medication-off states. Furthermore, Navratilova et al.'s research has also found that, at late motor complications stages, DBS treatment in PD patients exhibits a positive effect on gait compared to dopaminergic treatment alone. Regarding non-motor symptoms, various studies (Witjas et al., 2007; Jost et al., 2023) have posited that STN-DBS could induce improvements in diverse non-motor symptom types.

## Advancements in DBS research for PD

Emerging research in Deep Brain Stimulation (DBS) for Parkinson's Disease (PD) predominantly focuses on precise electrode localization strategies, stimulation device development, and programming. Surgical robot intertwines image-guided navigation and robotic arm technology, offering a suite of advantages such as automated coordinate adjustments, minimizing manual operation deviations, reducing human error, and shortening the operation time (Jin et al., 2020). Owing to advancements in MRI visualization, scholars suggest that surgical plans might be automated through machine learning, utilizing purely anatomical information provided by MRI (Shamir et al., 2019). The deployment of intraoperative MRI (iMRI) facilitates real-time electrode adjustments during procedures, augmenting accuracy over traditional methodologies and bolstering mid-term therapeutic outcomes (Ostrem et al., 2013).

To attenuate adverse reactions spurred by the quasi-spherical activation domain of ring contact stimulation, directional leads have been devised, showcasing improved clinical outcomes (Fricke et al., 2021). Notably, 32-contact electrodes have not only demonstrated superior therapeutic effects relative to traditional electrodes but also the adeptness to record local field potentials (LFPs) from other contacts during stimulation (Contarino et al., 2014). When synchronized with adaptive DBS systems (aDBS) (An et al., 2023), β-oscillate as a biomarker was used for negative feedback regulation of stimulus parameters. aDBS showed broad therapeutic prospects, which was only used in STN-DBS.

Efforts in postoperative programming predominantly focus on STN-DBS, especially observing the therapeutic implications of varied parameters, such as low frequency, short pulse width, and frequency conversion. These parameters are designed to enhance therapeutic outcomes while averting adverse reactions through the meticulous adjustment of activation domains across diverse contacts (Dayal et al., 2020). Moreover, the onset of imaging-based programming, together with activated tissue volume estimations, might suggest optimal contacts and therapeutic thresholds, potentially making programming more efficient and time-economical (Neudorfer et al., 2023).

## Outlook

DBS maintains its pivotal role as a principal surgical method for effectively treating PD. Advancing research into PD neural networks continues to deepen our understanding of the neuroelectrophysiological mechanisms underpinning the disease, enhancing electrode implantation precision and, thus, amplifying DBS efficacy. However, there is still a pressing need to mitigate side effects, further amplify long-term therapeutic outcomes, and elevate programming efficiency. Hence, adaptive programming (An et al., 2023) based on electrophysiological biomarkers and automated computerized symptom assessment and programming (Shamir et al., 2015) are anticipated to gain momentum, dependent on sustained interdisciplinary collaboration and continual clinical trials.

## Author contributions

ZL: Writing—original draft, Writing—review & editing. YZ: Writing—original draft, Writing—review & editing. YG: Writing—original draft, Writing—review & editing. XH: Writing—original draft, Writing—review & editing.
